# Leucine Improved Growth Performance, Muscle Growth, and Muscle Protein Deposition Through AKT/TOR and AKT/FOXO3a Signaling Pathways in Hybrid Catfish *Pelteobagrus vachelli × Leiocassis longirostris*

**DOI:** 10.3390/cells9020327

**Published:** 2020-01-30

**Authors:** Ye Zhao, Jin-Yang Li, Qin Jiang, Xiao-Qiu Zhou, Lin Feng, Yang Liu, Wei-Dan Jiang, Pei Wu, Jian Zhou, Juan Zhao, Jun Jiang

**Affiliations:** 1 College of Animal Science and Technology, Sichuan Agricultural University, Chengdu 611130, China; 2 Animal Nutrition Institute, Sichuan Agricultural University, Chengdu 611130, China; 3 Fish Nutrition and Safety Production University Key Laboratory of Sichuan Province, Sichuan Agricultural University, Chengdu 611130, China; 4 Fisheries Institute of Sichuan Academy of Agricultural Science, Chengdu 611731, China

**Keywords:** leucine, muscle growth, protein synthesis, protein degradation, hybrid bagrid catfish

## Abstract

(1) Background: l-leucine (Leu) plays a positive role in regulating protein turnover in skeletal muscle in mammal. However, the molecular mechanism for the effects of Leu on muscle growth and protein deposition is not clearly demonstrated in fish. This study investigated the effects of dietary Leu on growth performance and muscle growth, protein synthesis, and degradation-related signaling pathways of hybrid catfish (*Pelteobagrus vachelli♀ × Leiocassis longirostris♂*). (2) Methods: A total of 630 hybrid catfish (23.19 ± 0.20 g) were fed 6 different experimental diets containing graded levels of Leu at 10.0 (control), 15.0, 20.0, 25.0, 30.0, 35.0, and 40.0 g Leu kg^-1^ for 8 weeks. (3) Results: Results showed that dietary Leu increased percent weight gain (PWG), specific growth rate (SGR), FI (feed intake), feed efficiency (FE), protein efficiency ratio (PER), muscle fibers diameter, and muscle fibers density; up-regulated insulin-like growth factor I (IGF-I), insulin-like growth factor I receptor (IGF-IR), proliferating cell nuclear antigen (PCNA), myogenic regulation factors (MyoD, Myf5, MyoG, and Mrf4), and MyHC mRNA levels; increased muscle protein synthesis via regulating the AKT/TOR signaling pathway; and attenuated protein degradation via regulating the AKT/FOXO3a signaling pathway. (4) Conclusions: These results suggest that Leu has potential role to improve muscle growth and protein deposition in fish, which might be due to the regulation of IGF mRNA expression, muscle growth related gene, and protein synthesis and degradation-related signaling pathways. Based on the broken-line model, the Leu requirement of hybrid catfish (23.19-54.55 g) for PWG was estimated to be 28.10 g kg^-1^ of the diet (73.04 g kg^-1^ of dietary protein). These results will improve our understanding of the mechanisms responsible for muscle growth and protein deposition effects of Leu in fish.

## 1. Introduction

Aquaculture has become an agronomic activity with noticeable development around the world to respond to the increasing demand of aquatic products for human consumption [1]. The majority of fish growth is invested in accreting muscle tissue, which is the main edible portion [2,3]. Muscle growth is a complex, dynamic process involving both the recruitment of new muscle fibers (hyperplasia) and the growth of existing fibers (hypertrophy) [4], which is under the control of diverse regulatory factors such as insulin-like growth factors (IGFs), myogenic regulatory factors (MRFs), and myostatin (MSTN) [5]. The IGF-I is a key regulatory hormone that controls growth in vertebrates [6], which stimulates both proliferation and differentiation of myoblasts, as well as promoting myotube hypertrophy in vitro and in vivo [7,8,9]. The MRFs are muscle-specific basic helix–loop–helix transcription factors. They include myoblast determination protein (MyoD), myogenic factor 5 (Myf5), myogenin (MyoG), and myogenic regulatory factor 4 (MRF4) [10]. Myf5 and MyoD are mainly involved in muscle specification and trigger conversion of no muscle cells, such as fibroblasts, into muscle, whereas myogenin and MRF4 act later during myogenesis and allow myotube formation and maturation [11,12]. MSTN is a negative regulator of myogenesis, which inhibits myoblast cell proliferation and differentiation [13]. Despite increased understanding of the regulation of skeletal muscle growth by some of these factors, its regulation by nutrients remains poorly documented in fish. l-leucine (Leu) as a branched-chain amino acid is nutritionally essential for fish [14]. Leu deficiency results in depressed growth rate, low feed efficiency, and poor protein retention, as reported for large yellow croaker *Pseudosciaena crocea* [15], rainbow trout *Oncorhynchus mykiss* [16], juvenile golden pompano *Trachinotus ovatus* [17], grass carp *Ctenopharyngodon idellus* [18], black carp *Mylopharyngodon piceus* [14], fingerling channel catfish *Ictalurus punctatus* [19], juvenile hybrid grouper *Epinephelus fuscoguttatus*♀ × *Epinephelus lanceolatus*♂ [20], juvenile blunt snout bream *Megalobrama amblycephala* [21], *Catla catla* [22], and stinging catfish *Heteropneustes fossilis* [23]. Nevertheless, these studies mainly focused on the effects of Leu on growth, protein retention, and immunity. Recently, studies in primary preterm rat satellite cells and porcine myoblasts showed Leu promoted proliferation and differentiation [24,25]. Averous et al. (2012) also reported Leu deficiency inhibited the differentiation of both C2C12 myoblasts and primary mice satellite cells through regulating Myf5 and MyoD expression [26]. These data suggested that Leu might regulate muscle growth through affecting the process of cell proliferation and differentiation. However, the actual role of Leu in regulating muscle growth in fish still needs to be clarified.

The process involved in the increase in muscle growth is associated with accumulation of protein [27,28]. The protein deposition of muscle is the result of the balance of protein synthesis and degradation [29,30]. Previous studies have shown that nutrition can activate the IGF-I/PI3K/AKT signaling pathway and induce protein synthesis and accretion in rat and rainbow trout [31,32,33]. The target of rapamycin (TOR) is a downstream component of the PI3K/AKT pathway, which plays a crucial role in protein synthesis of fish [34]. The TOR regulates phosphorylation of its downstream effector ribosomal S6 kinase 1 (S6K1) and the eukaryotic translation initiation factor 4E-binding protein 1 (4E-BP1), ultimately promoting protein synthesis of fish [35,36,37,38]. Muscle protein degradation is primarily through the activation of the ubiquitin proteasome pathway (UPP), which can degrade most cell proteins and contribute to 75% protein degradation in muscle [39,40]. The AKT-dependent regulation of the forkhead box O3a (FOXO3a) protein has been shown to play a critical role in UPP pathway [41,42]. The AKT is known to phosphorylate FOXO3a, leading to the exclusion of phosphorylated FOXO3a proteins from the nucleus and the suppression of their transcriptional functions, which decreases muscle protein degradation in mammals [29,43,44]. Muscle atrophy F-box (MAFBX) and muscle Ring finger 1 (MURF-1) are responsible for increased protein degradation through the UPP pathway, which can actually be considered the master genes for muscle atrophy and wasting [39,45,46]. Leu could increase muscle protein deposition by regulating protein synthesis and protein degradation in mammals [29,47]. Dietary optimal Leu up-regulated liver TOR mRNA expression in juvenile hybrid grouper and juvenile blunt snout bream [20,21]. Supplementing media with Leu reduced protein degradation by regulating MAFBX32 expression in rainbow trout primary myocytes [48]. Those data suggested that Leu might elevate protein deposition by regulating gene expressions related to protein synthesis and protein degradation in fish. However, the evaluation of the effects of Leu on PI3K/AKT/TOR and AKT/FOXO3a pathways in vivo and their contribution to somatic growth have not been previously studied.

*Pelteobagrus vachelli*♀ × *Leiocassis longirostris*♂ is a hybrid catfish that has been widely cultured in China in recent years. Our previous studies have determined optimal dietary tryptophan levels [49]. Therefore, the objective of the present study was to investigate the effects of dietary Leu on growth performance and muscle growth, protein synthesis, and degradation-related signaling pathways of hybrid catfish. Furthermore, the dietary Leu requirement for hybrid catfish was evaluated.

## 2. Materials and Methods

### 2.1. Experimental Diets

Leu was obtained from Heng Yuan Biotech. Co. (Shanghai, China). The composition of the experimental diets is given in Table 1. Fish meal, casein, and gelatin were used as the main protein sources in all diets. Fish oil and soybean oil were used as the main lipid sources in all diets. The basal diet contained crude protein 384.7 g kg^−1^ and crude fat 71.0 g kg^−1^. Different concentrations of Leu were added to a basal diet mixture to constitute the seven levels of 10.0 (control group), 15.0, 20.0, 25.0, 30.0, 35.0, and 40.0 g Leu kg^−1^. A mixture of crystalline l-amino acids was supplemented to simulate the muscle essential amino acid pattern of hybrid catfish except for Leu (Table 2). All diets were made iso-nitrogenous by supplementation of l-glycine. The diets were prepared by mixing the dry ingredients with the oil using a mixer. Then, each diet was extruded in a twin-screw extruder (MY-165) with a 2 mm die. The processing conditions were as follows: 100 rpm screw speed, 127 °C temperature, and 30–45 atm pressure. Floating extruded pellets were air-dried and stored at 4 °C in plastic bags until use.

### 2.2. Feeding Management

Hybrid catfish were purchased from Rong Sen Corporation (Sichuan, China). Fish were adapted to the experimental environment for one month. A total of 630 fish with an average initial weight of 23.19 ± 0.20 g were randomly distributed into 21 tanks (200 × 100 × 105 cm^3^), resulting in 30 fish in each tank. Fish were fed with their respective diets to apparent satiation two times (7:00, 19:00) per day for 8 weeks at a feeding rate of about 3%–5% their weight. Average water temperature was 25 ± 2 °C. Dissolved oxygen was higher than 5 mg L^−1^ and pH was maintained at 7.0 ± 0.3. Continuous water flow was maintained at the rate of 1.2 L·min^−1^ in each tank. Fish were kept under a natural light and dark cycle. All experimental protocols were approved by the Animal Care Advisory Committee of Sichuan Agricultural University.

### 2.3. Sample Collection

After a fasting period of 24 h, all fish in each tank were weighed and counted at the initiation and termination of the feeding trail. Prior to sampling, 6 fish from each tank were anaesthetized in benzocaine bath (50 mg L^−1^). After slaughtering, fish were immediately filleted using a new sterile scalpel. Muscle samples from the left side of 6 fish in each tank were quickly frozen in liquid nitrogen and then stored at −80 °C until the determination of RNA content, gene expression, and Western blot. Muscle samples from the right side of the same fish were obtained for histological analysis.

### 2.4. Histological Analysis of Muscle

The right side of muscle samples for histochemical analyses were prepared according to the procedure as previously described [50]. Briefly, muscle was processed by dehydration in a graded series of ethanol solutions, followed by clearing in a series of xylenes, embedded in paraffin. Sections of 5 μm thickness were stained with hematoxylin and eosin and then prepared for light microscopy. The diameter and density of muscle fibers were measured by Image-Pro Plus 6.0 software. The outlines of about 150–200 muscle fibers were digitized from each block using Image Analysis System and fiber diameters were computed. About 1000–1500 muscle fibers were digitized per fish, distributed approximately equally between the different blocks. The fiber density was calculated as the number of fibers per mm^2^ of muscle cross-sectional area.

### 2.5. Real-Time Quantitative PCR

The total RNA was extracted from muscle with TRIZOL reagent (Invitrogen, Carlsbad, CA, USA) following the manufacturer's protocol. Total RNA quantity and quality were assessed by electrophoresis on 1% agarose gels and spectrophotometry at 260 and 280 nm. The 2 μL total RNA was used to synthesize cDNA using the PrimeScript® reverse transcription kit with gDNA Eraser (TaKaRa). Specific primers were designed according to the sequences cloned in our laboratory and the published sequences of hybrid catfish (Table 3). All of the real-time PCR analyses were performed in a CFX96 Real-Time PCR Detection System (Bio-Rad, Hercules, CA, USA). The amplification was performed in a 96-well plate in a 10 μL reaction volume containing 5 μL SYBR® PrimeScript™ RT-PCR Kit II (Bio-Rad), 0.5 μL (each) of the forward and reverse primer, 1 μL of template cDNA, and 3 μL of RNase-free water. The real-time PCR amplification was performed for each sample under following conditions: initial denaturation at 95 °C for 2 min, followed by 39 cycles of 95 °C/5 s, and optimal annealing temperature/30 s (Table 3). Target gene mRNA concentration was normalized to the mRNA concentration of the reference genes β-actin and 18S rRNA. The target and housekeeping gene amplification efficiency were calculated according to the specific gene standard curves generated from 10-fold serial dilutions. The expression results were calculated using the 2^−ΔΔC^_T_ method after verification that the primers amplified with an efficiency of approximately 100%.

### 2.6. Western Blotting

The processes for muscle protein extract preparation and Western blotting were conducted according to the procedures as previously described [51]. Briefly, the protein concentration was measured using a bicinchoninic acid (BCA) protein quantification Kit (Beyotime, Shanghai, China). Tissue lysates were separated by SDS–polyacrylamide gel and transferred to polyvinylidene difluoride membranes (Millipore, Massachusetts, USA). The membranes were blocked for 1 h at room temperature and then incubated with primary antibody overnight at 4 °C. Anti-phospho AKT (Ser473, #4060), anti-AKT (#4691), anti-phospho TOR (Ser2448, #2971), anti-TOR (#2972), anti-S6K1 (#9202), and anti-β-actin (#8457) were purchased from Cell Signaling Technology (Boston, Massachusetts, USA). Anti-phospho S6K1 (Thr421/Ser424, #380880), anti-MURF-1 (#503101), and anti-MAFBX (#503193) were purchased from Zen Biotechnology (Chengdu, Sichuan, China). Anti-phospho FOXO3a (Ser253, #ab154786) and anti-FOXO3a (#ab109629) were purchased from Abcam (Cambridge, London, UK). Antibody selection was performed in accordance with the method described by Skibacassy et al. [52]. Next, the membranes were washed in wash buffer three times for 10 min each, incubated with second anti-bodies for 1 h at room temperature, and then washed with buffer three times. Clarity western enhanced chemiluminescence substrate (Beyotime, Shanghai, China) was used to visualize signals. Densitometry analyses were performed following the method described by Liang et al. [53]. Different treatments were expressed relative to the level observed in the control group [54]. In addition, a ratio between the phosphorylated and total forms of the protein was calculated for each protein.

### 2.7. Calculations and Statistical Analysis

The results were represented as the means ± standard error of mean (SEM). The data were subjected to one-way analysis of variance, which was followed by Tukey’s method, to determine the significant differences among the groups by SPSS 20.0 (SPSS Inc., Chicago, IL, USA). *P* < 0.05 was considered to be statistically significant. Pearson correlation coefficient analysis was conducted using the Bivariate Correlation program. Dietary Leu requirement of hybrid catfish were estimated by the broken-line model.

## 3. Results

### 3.1. Effect of Dietary Leu on Growth Performance

**Figure 1:**
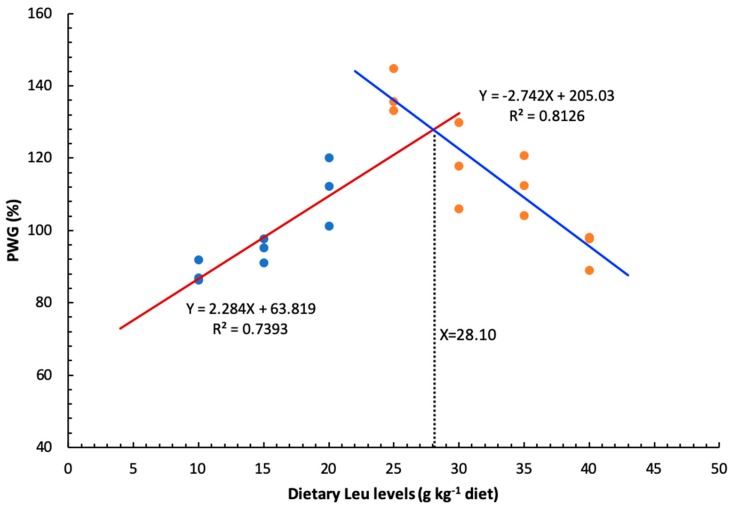
Broken-line analysis of PWG for hybrid catfish fed diets containing graded levels of Leu for 8 weeks.

As shown in Table 4, dietary Leu did not have a significant effect on the survival of hybrid catfish. The final body weight (FBW) was the highest for fish fed 25 g Leu kg^−1^ (*P* < 0.05), and no significant differences were found among other groups. The percent weight gain (PWG), specific growth rate (SGR), and feed efficiency (FE) were gradually increased for fish fed diets with increasing Leu levels up to 25 g kg^−1^, then gradually decreased (*P* < 0.05). The feed intake (FI) was the highest for fish fed the 25 g Leu kg^−1^ diet, and lowest for fish fed the 40 g Leu kg^−1^ diet (*P* < 0.05). The PER was highest for fish fed the 25 g Leu kg^−1^ diet, and lowest for fish fed the control diet (*P* < 0.05). Based on the broken-line model, the dietary Leu requirement of hybrid catfish for PWG was estimated to be 28.10 g kg^−1^ of the diet, corresponding to 73.04 g kg^−1^ of dietary protein (Figure 1).

### 3.2. Effects of Dietary Leu on Muscle Fiber Diameter and Density, Protein Content, and RNA/Protein Ratio

**Figure 2:**
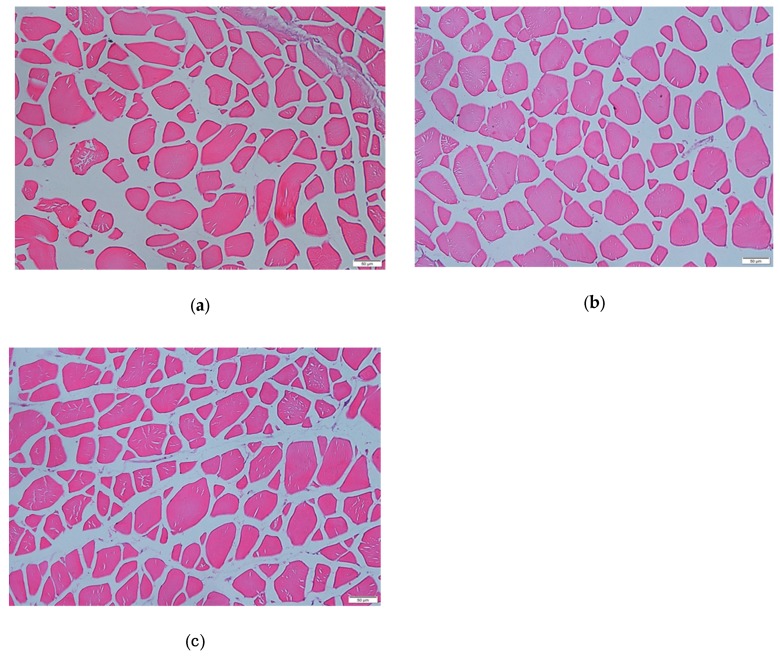
Microstructure of white muscle in cross-section (bars represent 50 μm; (**a**), 10.0 g Leu kg^−1^ diet group; (**b**), 25.0 g Leu kg^−1^ diet group; (**c**), 40.0 g Leu kg^−1^ diet group).

**Figure 3:**
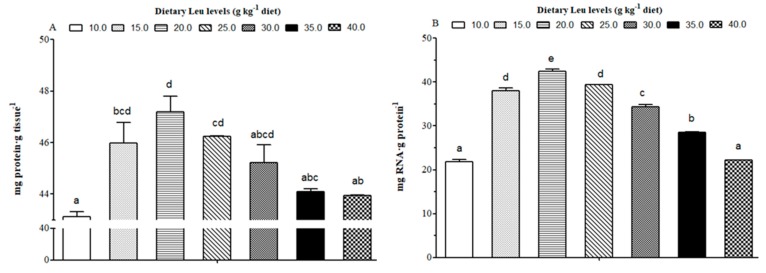
Effect of dietary Leu on protein content (mg protein·g tissue^−1^ (**A**)) and RNA/protein ratio (mg RNA·g protein^−1^ (**B**)) in hybrid catfish muscle. Data represent means ± SEM of three replicates, with six fish in each replicate. Values having different letters are significantly different (*P* < 0.05).

The microstructure of white muscle in cross-section is shown in Figure 2. The effects of dietary Leu on muscle fiber diameter and density are given in Table 5. The muscle fiber diameter gradually increased for fish fed diets with increasing Leu levels up to 25 g kg^−1^, and then gradually decreased (*P* < 0.05). The muscle fiber density was higher for fish fed Leu-supplemented diets than control diet (*P* < 0.05). The protein content (Figure 3A) and RNA/protein ratio (Figure 3B) gradually increased for fish fed diets with increasing Leu levels up to 20 g kg^-1^, and then gradually decreased (*P* < 0.05).

### 3.3. Effects of Dietary Leu on IGF-I and Muscle Growth Related Gene mRNA Expressions

**Figure 4:**
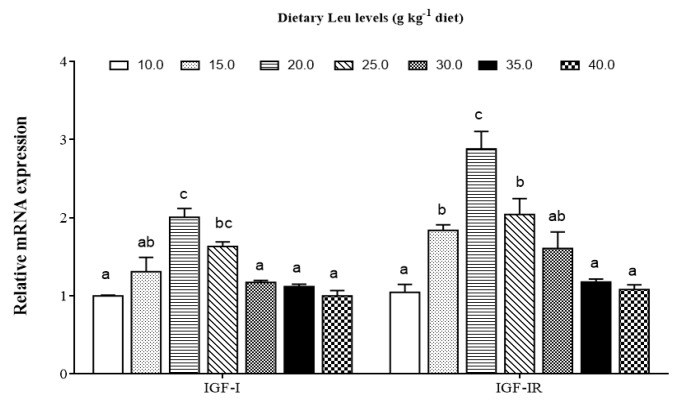
Effects of dietary Leu on IGF-I and IGF-IR gene expressions in muscle of hybrid catfish. Values are means ± SEM of three replicates, with six fish in each replicate, and different letters denote significant differences (*P* < 0.05).

**Figure 5:**
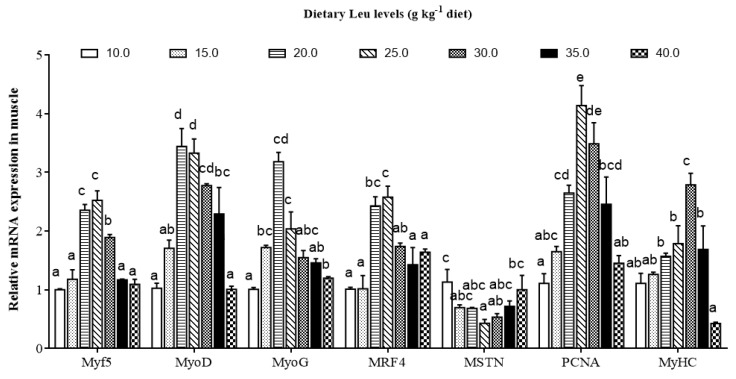
Effects of dietary Leu on muscle growth related gene mRNA expressions in muscle of hybrid catfish. Values are means ± SEM of three replicates, with six fish in each replicate, and different letters denote significant differences (*P* < 0.05).

As shown in Figure 4, the IGF-I and IGF-IR mRNA levels gradually increased for fish fed diets with increasing Leu levels up to 20 g kg^−1^, and decreased thereafter (*P* < 0.05). The effects of dietary Leu on muscle growth related gene mRNA expressions are presented in Figure 5. Fish fed diets containing 20.0, 25.0, and 30.0 g Leu kg^−1^ had higher mRNA level of Myf5 in muscle than those fish fed other diet groups (*P* < 0.05). The MyoD, MyoG, and PCNA mRNA expressions gradually increased with increasing Leu levels up to 20 g kg^−1^, and decreased thereafter (*P* < 0.05). In comparison with the control group, muscle mRNA level of MRF4 was higher in fish fed 20.0 and 25.0 g Leu kg^−1^ diets (*P* < 0.05). Fish fed 25 and 30 g Leu kg^−1^ diets had a lower level of MSTN mRNA expression than those fish fed the control diet (*P* < 0.05). The MyHC mRNA level was the highest for fish fed 30 g Leu kg^−1^ diet, followed by 20, 25, and 35 g Leu kg^−1^ diets, then control and 15 g Leu kg^−1^ diets, and the lowest for fish fed the 40 g Leu kg^−1^ diet (*P* < 0.05).

### 3.4. Effects of Dietary Leu on PI3K/AKT/TOR Signaling Pathway

**Figure 6:**
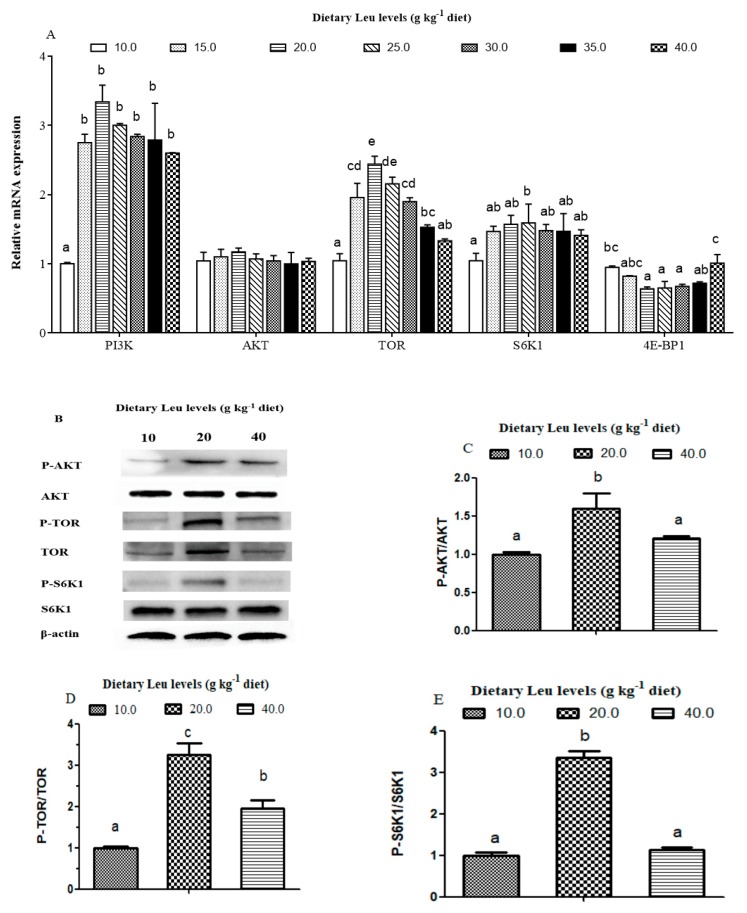
Effect of dietary Leu on the PI3K/AKT/TOR signaling pathway in hybrid catfish muscle. (**A**) Effect of dietary Leu on PI3K, AKT, TOR, S6K1, and 4E-BP1 mRNA expressions in muscle of hybrid catfish. (**B**) Effect of dietary Leu on the protein expressions of P-AKT (Ser473), AKT, P-TOR (Ser2448), TOR, P-S6K1 (Thr421/Ser424), and S6K1 in muscle of hybrid catfish. Results were expressed as the ratio of P-AKT and AKT (**C**), P-TOR and TOR (**D**), and P-S6K1 and S6K1 (**E**) protein levels. Data represent means ± SEM of three replicates, with six fish in each replicate. Values having different letters are significantly different (*P* < 0.05).

The effects of dietary Leu levels on protein synthesis related signaling molecule mRNA levels in hybrid catfish are presented in Figure 6A. The PI3K mRNA level was higher for fish fed Leu-supplemented diets than control diet (*P* < 0.05). No significant difference was observed in the AKT mRNA level among different dietary Leu levels. The TOR mRNA level in muscle was the highest for fish fed the 20 g Leu kg^−1^ diet, followed by the 25 g Leu kg^−1^ diet, then the 15 g Leu kg^−1^ diet, and the lowest for fish fed the control diet (*P* < 0.05). Fish fed 20, 25, and 30 g Leu kg^−1^ diets had lower levels of 4E-BP1 mRNA than those fish fed the control diet (*P* < 0.05). Fish fed diets containing 25.0 g Leu kg^−1^ had higher mRNA levels of S6K1 in muscle than those fish fed the control diet (*P* < 0.05).

The effects of dietary Leu levels on the AKT/TOR signaling pathway are presented in Figure 6B. There were no significant differences of the AKT and S6K1 protein levels among treatments. The protein level of TOR was higher for fish fed 20 g Leu kg^−1^ and 40 g Leu kg^−1^ diets than control diet (*P* < 0.05). The P-AKT, P-TOR, and P-S6K1 protein levels were higher for fish fed the 20 g Leu kg^−1^ diet compared with those fish fed 10 and 40 g Leu kg^−1^ diets (*P* < 0.05). Fish fed the 20 g Leu kg^−1^ diet had higher ratio of P-AKT and AKT (Figure 6C), P-TOR and TOR (Figure 6D), and P-S6K1 and S6K1 (Figure 6E) than those fish fed the control diet (*P* < 0.05).

### 3.5. Effects of Dietary Leu on Protein Degradation Related Signaling Pathway

**Figure 7:**
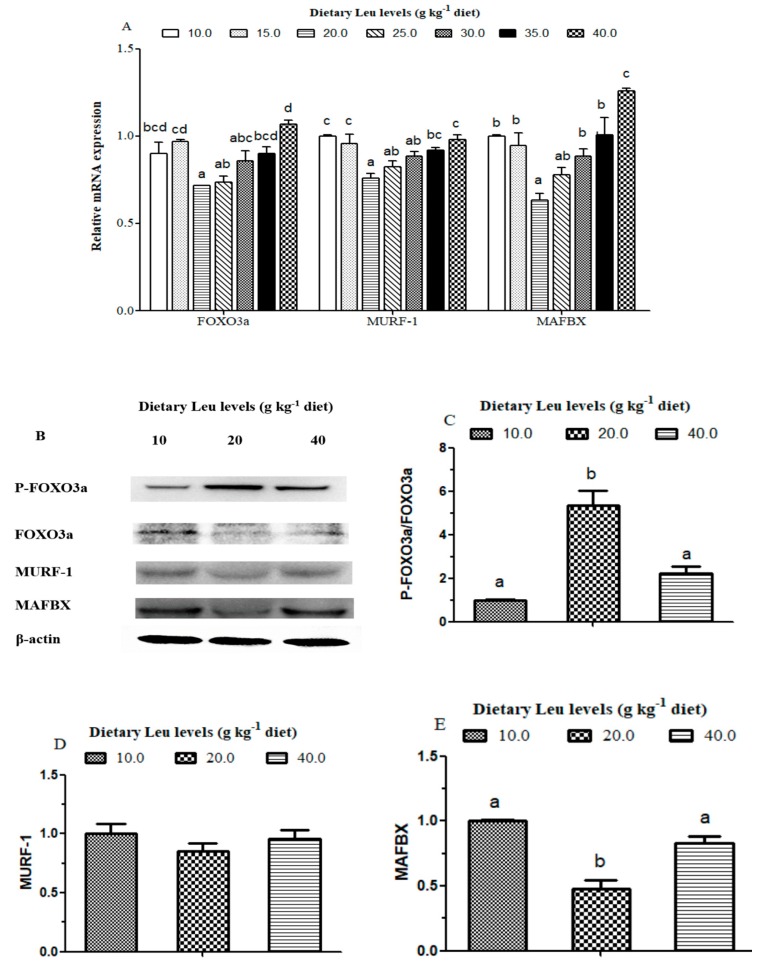
Effects of dietary Leu on the AKT/FOXO3a signaling pathway in hybrid catfish muscle. (**A**) Effect of dietary Leu on FOXO3a, MURF-1, and MAFBX mRNA expressions in muscle of hybrid catfish. (**B**) Effect of dietary Leu on the protein expressions of P-FOXO3a (Ser253), FOXO3a, MURF-1, and MAFBX in muscle of hybrid catfish. Results were expressed as the ratio of P-FOXO3a and FOXO3a (**C**), MURF-1 (**D**), and MAFBX (**E**) protein levels. Data represent means ± SEM of three replicates, with six fish in each replicate. Values having different letters are significantly different (*P* < 0.05).

The effects of dietary Leu levels on protein degradation related signaling molecule mRNA levels are presented in Figure 7A. The FOXO3a mRNA level was the lowest in muscle for fish fed the 20 g Leu kg^−1^ diet, and the highest for fish fed the 40 g Leu kg^−1^ diet (*P* < 0.05). The mRNA levels of MURF-1 and MAFBX gradually decreased for fish fed diets with increasing Leu levels up to 20 g kg^−1^, and increased thereafter (*P* < 0.05).

The effects of dietary Leu levels on protein degradation related signaling molecule protein expressions are presented in Figure 7B–E. The FOXO3a and MAFBX protein levels were lower for fish fed the 20 g Leu kg^−1^ diet compared with those fish fed 10 and 40 g Leu kg^−1^ diets (*P* < 0.05). The P-FOXO3a protein level was higher for fish fed the 20 g Leu kg^−1^ diet than those fish fed 10 and 40 g Leu kg^-1^ diets (*P* < 0.05). Fish fed the 20 g Leu kg^−1^ diet had higher a ratio of P-FOXO3a and FOXO3a than those fish fed the control diet (*P* < 0.05). However, no significant difference in the MURF-1 protein level among treatments was detected (*P* > 0.05).

## 4. Discussion

As an essential amino acid, dietary Leu level had a clear effect on growth and feed utilization of hybrid catfish. The optimal dietary Leu requirement for maximal PWG of hybrid catfish was estimated to be 28.10 g kg^−1^ of the diet, corresponding to 73.04 g kg^−1^ of dietary protein. This value (g kg^−1^ of dietary protein) was similar to that of large yellow croaker (67.90 g kg^−1^ of dietary protein) [15], lower than those for rainbow trout (92.00 g kg^−1^ of dietary protein) [16] and juvenile golden pompano (80.20 g kg^−1^ of dietary protein) [17], but higher than those reported for grass carp (42.40 g kg^−1^ of dietary protein) [18], black carp (59.50 g kg^−1^ of dietary protein) [14], fingerling channel catfish (35.00 g kg^−1^ of dietary protein) [19], juvenile hybrid grouper (47.50 g kg^−1^ of dietary protein) [20], juvenile blunt snout bream (47.40 g kg^−1^ of dietary protein) [21], *Catla catla* (47.90 g kg^−1^ of dietary protein) [22], and stinging catfish (43.40 g kg^−1^ of dietary protein) [23]. The wide variation (35.00–92.00) observed in the requirements for Leu among fish species may be due to the differences in dietary protein sources, size and age of fish, genetic and species differences, feeding practices, or growth environment. Furthermore, the hybrid catfish fed diets with Leu levels above 30 g kg^−1^ diet resulted in a significant reduction in growth. Similarly, excess Leu in the diet also appeared to have adverse effects on growth of other fish species [55,56,57], and this was attributed to the possible antagonism between Leu and other branched chain amino acid [58]. A previous study also demonstrated that antagonism between branched chain amino acids generally arises in animals from an excess of Leu intake over isoleucine and valine because the requirements of branched chain amino acids were affected by each other [55]. On the other hand, the supplementation of valine in excessive Leu diets could relieve the depression of growth and feed utilization in Lake trout *Salvelinus namaycush* [59].

Skeletal muscle is the main part of the trunk of fish, accounting for about 40%–60% of body weight. Most fish muscle is composed of white muscle [60]. Fish muscle fiber is the basic unit of skeletal muscle, which represents the development of muscle [61]. The changes in diameter and density of white muscle has a certain guiding role in understanding the growth and development process of fish muscles. The nutritional status has been shown to affect muscle mass in mammals and teleosts [62,63]. The present results for the first time show that dietary Leu significantly increased the muscle fiber diameter and density in fish, indicating that Leu has beneficial effects on muscle growth and development, which were in accordance with the result for C2C12 [64]. The development of fish skeletal muscle is modulated by IGFs [65]. Our data indicated that relative expressions of IGF-I and IGF-IR genes were up-regulated by Leu. Similarly, a study in gilthead sea bream cultured myocytes demonstrated that Leu deficiency significantly reduced IGF-I mRNA expression [66]. The previous study observed Leu increased skeletal muscle IGF-I concentration in resistance-trained men [67]. Zhou et al. also demonstrated that dietary optimal Leu elevated liver IGF-I mRNA expression [20]. Fish muscle growth is a complicated and precisely controlled process, including proliferation and differentiation of myoblasts [68]. The IGF-I stimulates both proliferation and differentiation of myoblasts [69] to promote muscle growth in fish [65,70]. Correlation analysis indicated that the IGF-I was positively correlated with muscle fiber diameter (r = 0.724, *P* = 0.066, Table 6), indicating that dietary Leu increasing muscle fiber hypertrophy in fish might be partly related to up-regulated IGF-I transcription. Moreover, skeletal muscle is generated from satellite cells that, when activated, proliferate, fuse, and differentiate to form new myofibers. This process is also regulated by several myogenic regulatory factors [1,61,71]. During fish muscle growth, PCNA is a marker of proliferation expressed in activated satellite cells [72]. The MyoD and Myf5 regulate the activation and proliferation of satellite cells, whereas myogenin and MRF4 act on cell differentiation [73,74]. The MyHC plays important roles in fish muscle growth via hyperplasia and hypertrophy of muscle fibers [75]. The present results showed that dietary Leu up-regulated PCNA, Myf5, MyoD, MRF4, MyoG, and MyHC mRNA expressions in fish. This result was in good agreement with reports on C2C12 myoblasts, preterm rat satellite cells, and porcine myoblasts. These studies reported that Leu limitation prevents the differentiation of myoblasts and primary satellite cells [24,25,26]. Chen et al. also demonstrated that Leu could promote proliferation of C2C12 myoblasts [76]. Correlation analysis indicated that IGF-I was positively correlated with Myf5 (r = 0.837, *P* = 0.019), MyoD (r = 0.820, *P* = 0.024), MyoG (r = 0.977, *P* = 0.000), and MRF4 (r = 0.768, *P* = 0.044) mRNA levels (Table 6), suggesting that dietary Leu increasing the muscle growth in fish might be partly related to up-regulated IGF-I transcription. MSTN, a member of the transforming growth factor-β superfamily, is expressed predominantly in skeletal muscle of fish, and the downstream function is to prevent the progression of myogenic cells into the cell division cycle [77]. The present study showed dietary optimal Leu down-regulated MSTN mRNA expression in hybrid catfish. The result is similar to the finding in C2C12 myoblasts [78]. The decrease of MSTN gene expression in the muscle with Leu might be related to miR-27a. Previous studies demonstrated that Leu induced proliferation promotion through miR-27a-mediated regulation of the MSTN in C2C12 cells [76,79]. Recently, growing evidence from fish suggests that muscle growth is regulated by essential amino acids [80,81,82]. Alami-Durante et al. reported dietary methionine affected the expression of genes regulating specific transition points of myogenesis and the expression of muscle structural genes and growth factors involved in satellite cell activation and muscle growth [81]. Michelato et al. reported that dietary histidine upregulated MyoD and MyoG mRNA expression and affected muscle hyperplasia of Nile tilapia juveniles *Oreochromis niloticus* [80]. Childress et al. also demonstrated that lysine supplementation of commercial fishmeal-free diet regulated MSTN and MyoG mRNA expression and controlled myogenesis in hybrid striped bass *Morone chrysops × M. saxatilis* [82]. These results suggested essential amino acids plays a critical role in regulating muscle growth. However, more studies are required to elucidate a more detailed mode in which Leu regulates muscle growth related gene expression in fish.

Leu is considered to be an important nutrition regulation factor involved in protein synthesis in muscle. The tissue RNA/protein ratio could reflect the capacity for protein synthesis in fish [83]. In the present study, dietary Leu improved protein content and the RNA/protein ratio of muscle. The PI3K/AKT signaling pathway plays a crucial role in protein synthesis [84,85,86]. The AKT phosphorylation is an important marker of the activation of the PI3K/AKT pathway [8]. Despite their significance, however, their regulation by nutrients remains poorly understood in fish. The present study demonstrated dietary Leu supplementation resulted in up-regulation of PI3K mRNA levels. The phosphorylation level and phospho-total ratio of AKT were elevated in fish fed the 20 g Leu kg^−1^ diet. The correlation analysis also indicated that muscle protein content was positively correlated with PI3K (r = 0.782, *P* = 0.038) and AKT (r = 0.822, *P* = 0.023) mRNA levels (Table 6). These results indicated that the PI3K/AKT signaling pathway contributed to Leu-induced muscle protein synthesis. Meanwhile, the present results also showed IGF-I was positively correlated with mRNA expressions of PI3K (r = 0.637, P= 0.124) and AKT (r = 0.855, *P*= 0.014), which suggested that dietary Leu promotes muscle protein synthesis by activating the PI3K/AKT signaling pathway via IGF-I. Similar results were observed in muscle of rainbow trout and fine flounder [33,87]. The TOR signaling pathway, a downstream component of the PI3K/AKT pathway, is necessary for stimulating translation initiation and enhancing muscle protein synthesis. Activated TOR, which has a major role in mRNA translation, promotes 4E-BP1 and S6K1 phosphorylation in fish [88,89]. Once phosphorylated, eukaryotic initiation factor 4E (eIF4E) is released by 4E-BP1 and then becomes the active eIF4G·eIF4E complex, which binds to mRNA and initiates translation. Phosphorylated S6K1 also takes part in translation initiation [90]. The present data showed that dietary Leu supplementation increased muscle TOR and S6K1 mRNA levels and the phosphorylation of TOR and S6K1. The correlation analysis also indicated that muscle protein content was positively correlated with TOR (r = 0.987, *P*= 0.000) and S6K1 (r = 0.791, *P*= 0.034) mRNA levels, and negatively correlated with 4E-BP (r = −0.730, *P*= 0.062) mRNA level (Table 6), indicating that dietary Leu increasing muscle protein content might be partly related to elevate muscle protein synthesis via the TOR signaling pathway in fish. In mammals, Leu was proved to modulate protein synthesis directly through mTOR or indirectly through PI3K/AKT/mTOR signaling pathways [91,92,93]. Lang et al. reported that TOR phosphorylation induced by Leu could be independent of the PI3K/AKT signaling pathway [94]. These findings therefore suggest that in teleosts, as in mammals, Leu could activate the TOR or PI3K/AKT/TOR signaling pathways and contribute to muscle protein synthesis in fish. However, more studies are required to elucidate a more detailed mode in which Leu improves muscle protein synthesis in fish.

Protein degradation in mammals mainly occurs through two intracellular proteolytic systems: proteasomes and lysosomes [95]. Most of the protein in skeletal muscle can be degraded via the UPP pathway [39]. The AKT also appears to play a key role in protein degradation pathways, which phosphorylates and inactivates the FOXO family of transcription factors, promoting the export of FOXOs from the nucleus to the cytoplasm. The activation of AKT and inactivation of FOXO would down-regulate the activity of the UPP pathway, such as MAFBX and MURF-1 [39], and consequently result in a decrease in protein degradation [29,96]. Several reports in mammals reported that Leu suppressed muscle protein degradation by the inhibition of the UPP pathway [97,98,99]. In the present study, the mRNA levels of FOXO3a, MAFBX, and MURF-1 decreased for fish fed diets with increasing Leu levels up to 20 g kg^−1^, suggesting that Leu may inhibit protein degradation by the UPP pathways. As expected, Western blotting results showed the phosphorylation level and phospho-total ratio of FOXO3a were elevated in fish fed the 20 g Leu kg^−1^ diet. The protein levels of MAFBX were decreased in fish fed the 20 g Leu kg^-1^ diet. The previous study on rainbow trout primary myocytes showed Leu reduced protein degradation by regulating MAFBX32 mRNA expression [48]. The correlation analysis also indicated that muscle protein content was negatively correlated with FOXO3a (r = −0.649, *P*= 0.115), MURF-1 (r = −0.838, *P*= 0.019), and MAFBX (r = −0.808, *P*= 0.028) mRNA levels (Table 6), which suggested that the dietary Leu attenuated protein degradation was partly related to AKT/FOXO3a signaling pathway. To our best knowledge, this is the first report demonstrating that Leu could promote protein deposition by preventing protein degradation via the AKT/FOXO3a signaling pathway in fish. In addition, increasing evidence indicated that IGF-I could inhibit overall protein breakdown, degradation of myofibrillar proteins, and expression of MURF-1 [100,101]. Correlation analysis indicated that IGF-I was negatively correlated with FOXO3a (r = −0.809, *P* = 0.028), MURF-1 (r = −0.923, *P* = 0.003), and MAFBX (r = −0.883, *P*= 0.008) mRNA levels (Table 6), indicating that dietary Leu decreasing the muscle protein degradation in fish might be partly related to up-regulated IGF-I transcription. Taken together, these data indicated that Leu prevented protein degradation via the IGF-I/AKT/FOXO3a signaling pathway. To our knowledge, no study has focused on the effects of Leu on protein degradation related to the IGF-I/AKT/FOXO3a signaling pathway in fish.

## 5. Conclusions

In summary, the present work showed that Leu improved growth performance of hybrid catfish. In addition, dietary Leu improved muscle protein synthesis in hybrid catfish by activating the AKT/TOR signaling pathway. For the first time, we also found that dietary Leu decreased muscle protein degradation via the AKT/FOXO3a signaling pathway. These results provided partial theoretical evidence for the improvement of muscle growth and protein deposition by Leu in fish. Lastly, based on the broken-line model, the dietary requirement of hybrid catfish (23.19–54.55 g) for PWG was estimated to be 28.10 g kg^−1^ of the diet, corresponding to 73.04 g kg^−1^ of dietary protein.

## Figures and Tables

**Table 1:** Composition and nutrients content of basal diet (g kg^−1^).

Ingredients		Nutrients content^a^	
Fish meal	170.0	Crude protein	384.7
Casein	10.0	Crude fat	71.0
Gelatin	10.0	Available phosphorus	14.8
Amino acid premix^b^	170.0	ω-3	15.2
Leucine-glycine premix^c^	120.0	ω-6	13.9
α-starch	210.0		
Corn starch	168.3		
Fish oil	30.0		
Soybean oil	26.0		
Vitamin premix^d^	10.0		
Mineral element premix^e^	20.0		
Ca(H_2_PO_4_)_2_	40.0		
Choline chloride (50%)	10.0		
Ethoxyquin (30%)	0.5		
Cellulose	5.0		
XanthophyII	0.2		

^a^ The values of crude protein, crude fat, and crude ash were measured. Available phosphorus, n-3 and n-6 contents calculated according to NRC (2011). ^b^ Amino acid mix (g kg^−1^): lysine, 125.204; histidine, 27.508; isoleucine, 39.425; arginine, 116.491; methionine, 60.071; cystine, 11.432; phenylalanine, 77.364; threonine, 83.764; tryptophan, 11.723; valine, 11.302; all ingredients were diluted with corn starch to 1 kg. ^c^ Leucine–glycine premix composition from diet 1 to 7 was as follows (g kg^−1^): l-leucine 0.0, 41.7, 83.3, 125.0, 166.7, 208.3, 250.0; glycine 152.8, 127.3, 101.9, 76.3, 50.9, 25.5, 0.0; corn starch 847.2, 831.0, 814.8, 798.7, 782.0, 766.2, 750.0, respectively. ^d^ Vitamin premix (g kg^−1^): retinyl acetate (500,000 IU g^−1^), 8.063; cholecalciferol (500,000 IU g^−1^), 0.100; dl-α-tocopherol acetate (500 g kg^−1^), 53.600; menadione (230 g kg^−1^), 0.217; cyanocobalamin (10 g kg^−1^), 0.100; d-biotin (20 g kg^−1^), 5.000; folic acid (960 g kg^−1^), 0.521; thiamin nitrate (900 g kg^−1^), 0.111; ascorbyl acetate (930 g kg^−1^), 86.022; niacin (990 g kg^−1^), 3.143; mesoinositol (990 g kg^−1^), 52.323; calcium-d-pantothenate (900 g kg^−1^), 1.667; riboflavine (800 g kg^−1^), 1.125; pyridoxine hydrochloride (810 g kg^−1^), 0.370; all ingredients were diluted with corn starch to 1 kg. ^e^ Mineral premix (g kg^−1^): FeSO4·H2O (300 g kg^−1^ Fe), 13.333; CuSO4·5H2O (250 g kg^−1^ Cu), 1.300; ZnSO4·H2O (345 g kg^−1^ Zn), 13.043; MnSO4 ·H2O (318 g kg^−1^ Mn), 4.717; KI (38 g kg^−1^ I), 1.447; NaSeO3 (10 g kg^−1^ Se), 1.000; MgSO4·H2O (150 g kg^−1^ Mg), 133.333. All ingredients were diluted with CaCO3 to 1 kg.

**Table 2:** Amino acid composition of the experimental diets (% dry diet) and fish muscle (relative to 384.7 g kg^−1^ protein)^1^

Amino Acid	Dietary Leu Level (g kg^−1^ Diet)							384.7 g kg^−^^1^ Muscle Protein
	10	15	20	25	30	35	40	
Essential amino acid								
Threonine	1.93	1.89	1.95	1.94	1.90	1.87	1.93	1.94
Valine	1.34	1.38	1.41	1.36	1.39	1.42	1.43	1.40
Methionine	1.26	1.25	1.19	1.23	1.23	1.25	1.22	1.21
Isoleucine	1.17	1.14	1.12	1.16	1.20	1.18	1.17	1.18
Leucine	1.11	1.48	2.02	2.52	3.04	3.57	4.02	2.53
Phenylalanine	1.81	1.79	1.80	1.82	1.75	1.83	1.76	1.77
Histidine	0.84	0.76	0.80	0.82	0.79	0.81	0.85	0.83
Lysine	2.61	2.59	2.62	2.57	2.65	2.58	2.55	2.62
Arginine	2.57	2.55	2.47	2.60	2.58	2.61	2.49	2.53
Nonessential amino acid								
Aspartic acid	4.41	4.50	4.62	4.55	4.48	4.68	4.60	4.56
Serine	1.91	1.93	1.87	1.95	2.01	1.94	2.05	1.97
Glutamic acid	6.82	6.77	6.68	6.71	6.75	6.69	6.66	6.62
Glycine	2.22	2.28	2.26	2.30	2.32	2.29	2.25	2.21
Alanine	2.73	2.85	2.80	2.78	2.71	2.90	2.87	2.82
Cystine	0.21	0.20	0.19	0.25	0.18	0.21	0.17	0.23
Tyrosine	1.51	1.51	1.47	1.45	1.55	1.56	1.49	1.50
Proline	1.44	1.48	1.51	1.57	1.48	1.50	1.53	1.46

^1^ Values are means from duplicate samples of experimental diets and fish muscle.

**Table 3:** Primer sequences and optimal annealing temperatures (OATs) of genes selected for analysis by real-time PCR.

Name	Sequence (5′–3′)	OAT (°C)	Accession Number
Myf5-QF	CTCCAGTCCTTCATCATCCACC	64.9	MK253547
Myf5-QR	CACTCGCACTCTGACCTTCGT		
MyoD-QF	CCTAATCAGAGGCTTCCCA	55.5	HM363525
MyoD-QR	TCACCGCTGTATTGTTCCA		
MyoG-QF	TACTTTTTCCCCGAACAGC	57.6	HQ246723
MyoG-QR	TCCAGTCCTACATTGCCAGA		
MRF4-QF	CAGACTGTCAGAGGACGGGG	52.8	MK281342
MRF4-QR	CAGCCTTCTCTTTGGTGGGA		
MSTN-QF	ACGCCACTACCGAGACCG	64.6	DQ767967
MSTN-QR	CTCAATACCCCAGTTTGTTTCC		
PCNA-QF	GTTGATGGACTTGGATGTGGA	60.1	MK281343
PCNA-QR	CGTTGCTGGTTTGGGAGA		
MyHC-QF	GCAATGAAGGAGAACTATG	60.0	MK440319
MyHC-QR	TCACACTTTCCTCAGCGT		
IGF-I-QF	ATCTGGGTAATGTGACTGCCGA	56.8	KX434878
IGF-I-QR	TTCATCATCTCCGCCCTTGC		
IGF-IR-QF	ACACCGATGAGGGAAACTGG	56.6	MG773202
IGF-IR-QR	GTGGATGAAGGACGGGAACA		
PI3K-QF	GTGAATGGGAAAGACGCT	62.6	MG773208
PI3K-QR	GCACACAGGACTCCAGATGA		
AKT-QF	TCTACCCTTTACACCTGCTGAC	61.7	KX131157.1
AKT-QR	GATGGCTGGGATTGCTTTC		
TOR-QF	GACAAACGGAGGAAGGAGG	58.2	MG773199
TOR-QR	TCATCAGGAAAGAAGAGGGACT		
4E-BP1-QF	ACGCCACCCAGTTGCCTA	62.6	MG773207
4E-BP1-QR	GGATGCTTTTGCTGCCGAC		
S6K1-QF	GCAAACTGAATCTCCCACCC	61.7	MG773195
S6K1-QR	AGGCTTGAAAGGCGGCTC		
MURF-1-QF	CCGTTTTGAGGTGGTGCT	53.6	MK756118
MURF-1- QR	TGTTCTCCAGTTGTTGCTTGTA		
MAFBX-QF	AACCTCTGTCACTACCACTTCACT	54.8	MK812970
MAFBX- QR	GGTCGCTGTACTGCTCTTTATG		
FOXO3a-QF	GACTTCCGCTCTCGCACTAA	60.5	MK562423
FOXO3a-QR	ATCATCAGCAACCTCATCCACT		
β-actin-QF	CCTAAAGCCAACAGGGAAAA	59	EU161066
β-actin-QR	ATGGGGCAGAGCATAACC		
18S-QF	CCTGAGAAACGGCTACCACATCC	57.1	KP938527
18S-QR	AGCAACTTTAATATACGCTATTGGAG		

**Table 4:** Initial body weight (IBW, g fish^-1^), survival, final body weight (FBW, g fish^-1^), percent weight gain (PWG, %), specific growth rate (SGR, %/d), feed intake (FI, g fish^-1^), feed efficiency (FE, %), and protein efficiency ratio (PER) of hybrid catfish fed diets containing graded levels of Leu (g kg^-1^) for 8 weeks.

Leu	10.0	15.0	20.0	25.0	30.0	35.0	40.0
IBW	23.21 ± 0.13	22.98 ± 0.14	22.90 ± 0.20	23.19 ± 0.27	23.32 ± 0.24	23.30 ± 0.25	23.34 ± 0.19
Survival	96.67 ± 1.93	96.67 ± 0.00	95.56 ± 2.94	98.89 ± 1.11	95.56 ± 1.11	97.78 ± 2.22	94.44 ± 2.93
FBW	43.73 ± 0.70^a^	44.73 ± 0.13^a^	48.22 ± 1.32^a^	54.55 ± 0.87^b^	49.48 ± 1.58^ab^	47.82 ± 2.22^a^	45.53 ± 1.17^a^
PWG	88.35 ± 1.81^a^	94.70 ± 1.95^ab^	111.20 ± 5.51^ab^	137.89 ± 3.56^c^	117.91 ± 11.96^bc^	112.42 ± 8.26^ab^	94.94 ± 2.99^ab^
SGR	1.13 ± 0.02^a^	1.19 ± 0.02^a^	1.33 ± 0.05^ab^	1.55 ± 0.03^b^	1.34 ± 0.07^ab^	1.27 ± 0.08^a^	1.19 ± 0.03^a^
FI	37.57 ± 1.19^bc^	38.60 ± 0.54^bc^	42.04 ± 1.84^c^	43.30 ± 3.74^c^	38.00 ± 0.57^bc^	34.25 ± 1.02^ab^	31.66 ± 1.74^a^
FE	53.05 ± 1.42^a^	56.87 ± 2.79^a^	59.08 ± 1.15^a^	74.87 ± 2.67^b^	71.85 ± 4.15^b^	69.01 ± 4.27^b^	67.34 ± 1.48^b^
PER	2.06 ± 0.03^a^	2.09 ± 0.07^a^	2.41 ± 0.03^ab^	3.11 ± 0.37^b^	2.53 ± 0.21^ab^	2.52 ± 0.08^ab^	2.58 ± 0.01^ab^
Regressions							
Y_PWG_ = -0.1535X^2^ + 8.1194X + 16.5380					X = 26.45	R^2^ = 0.7833	*P* = 0.047
Y_SGR_ = -0.0012X^2^ + 0.0647X + 0.5707					X = 26.96	R^2^ = 0.7366	*P* = 0.075
Y_FI_ = -0.03199X^2^ + 1.382X + 26.57					X = 21.60	R^2^ = 0.8765	*P* = 0.015
Y_FE_ = -0.043X^2^ + 2.7213X + 27.73					X = 31.65	R^2^ = 0.7959	*P* = 0.042
Y_PER_ = -0.0019X^2^ + 0.1148X + 1.0029					X = 30.21	R^2^ = 0.5735	*P* = 0.182

Values are means ± SEM (n = 3, 30 fish in each replicate). Mean values with different superscripts in the same row are significantly different (*P* < 0.05). PWG =weight gain (g) / initial weight (g) × 100; SGR = (ln FBW-ln IBW)/d× 100; FE = weight gain (g) / feed intake (g) × 100; PER = weight gain (g) / protein intake (g).

**Table 5:** The muscle fiber diameter (DI, μm) and density (DE, n/mm^2^) of hybrid catfish fed diets containing graded levels of Leu (g kg^-1^) for 8 weeks.

Leu	10.0	15.0	20.0	25.0	30.0	35.0	40.0
DI	28.85 ± 0.42^a^	29.36 ± 0.92^a^	34.35 ± 2.92^ab^	38.48 ± 1.85^b^	33.31 ± 1.64^ab^	29.74 ± 0.31^a^	28.63 ± 0.77^a^
DE	64.00 ± 1.79^a^	79.4 ± 3.97^b^	88.4 ± 4.53^bc^	103.5 ± 7.58^c^	90.6 ± 2.5^bc^	95.4 ± 1.81^bc^	87 ± 6.82^bc^

Values are means ± SEM (n = 3, 18 fish per treatment). Mean values with different superscripts in the same row are significantly different (*P* < 0.05).

**Table 6:** Correlation analysis of parameters in the muscle of hybrid catfish.

Independent Parameters	Dependent Parameters	Correlation Coefficients	*P*
PWG	FE	0.796	0.032
	PER	0.872	0.011
IGF-I mRNA	Muscle fiber diameter	0.724	0.066
	Myf5 mRNA	0.837	0.019
	MyoD mRNA	0.820	0.024
	MyoG mRNA	0.977	0.000
	MRF4 mRNA	0.768	0.044
	PI3K mRNA	0.637	0.124
	AKT mRNA	0.855	0.014
	TOR mRNA	0.897	0.006
	4E-BP mRNA	-0.703	0.078
	S6K1 mRNA	0.639	0.122
	FOXO3a mRNA	-0.809	0.028
	MURF-1 mRNA	-0.923	0.003
	MAFBX mRNA	-0.883	0.008
Protein content	IGF-I mRNA	0.912	0.004
	Myf5 mRNA	0.801	0.031
	MyoD mRNA	0.803	0.030
	MyoG mRNA	0.902	0.005
	MRF4 mRNA	0.670	0.099
	MSTN mRNA	-0.736	0.059
	PI3K mRNA	0.782	0.038
	AKT mRNA	0.822	0.023
	TOR mRNA	0.987	0.000
	4E-BP mRNA	-0.730	0.062
	S6K1 mRNA	0.791	0.034
	FOXO3a mRNA	-0.649	0.115
	MURF-1 mRNA	-0.838	0.019
	MAFBX mRNA	-0.808	0.028
TOR mRNA	Myf5 mRNA	0.840	0.018
	MyoD mRNA	0.876	0.010
	MyoG mRNA	0.895	0.006
	MRF4 mRNA	0.713	0.072
	MSTN mRNA	-0.814	0.026
	PCNA mRNA	0.672	0.098
4E-BP mRNA	Myf5 mRNA	-0.806	0.029
	MyoD mRNA	-0.963	0.000
	MyoG mRNA	0.697	0.082
	MRF4 mRNA	-0.647	0.116
	MSTN mRNA	0.887	0.008
	PCNA mRNA	-0.851	0.015
	MyHC mRNA	0.801	0.030
S6K1 mRNA	Myf5 mRNA	0.677	0.095
	MyoD mRNA	0.760	0.047
	MyoG mRNA	0.663	0.105
	MRF4 mRNA	0.698	0.081
	MSTN mRNA	-0.839	0.018
	PCNA mRNA	0.706	0.076
